# Monthly Summary

**Published:** 1898-02

**Authors:** 


### Monthly Summary.

GOLD FILLINGS INSERTED WHILE CAVITY FILLED WITH SALIVA.—Mr. R. Heide, Professor of the Ecole Dentaire of Paris, had the pleasure to submit, at a meeting of Odontological Society, February 2nd, 1897, teeth filled by Dr. Herbst with sub-marine gold. Sub marine gold is a special gold 60 in thickness, therefore very thick, but nevertheless very soft and malleable; it is put in the form of pellets. With this kind of gold not only central cavities can be filled, but proximal cavities also, providing that the walls are very strong, not recommended for building loose part of tooth, such as corners, etc. Instruments used for the working of this gold are simple. First, a large polishing bur; second, same as first but unpolished; two pluggers for soft gold, this will constitute the whole. *Modus faciendi*—introduce large pellets of sub-marine gold with pluggers, then use bur No. 1, when in place fit in the gold, adding more gold till the cavity is filled. It is known for a long time the way of excluding humidity caused by saliva, by the use of the rubber-dam, but before these operators were subject to many inconveniences on account of the saliva, rendering work very tedious, and in many cases the work would not be finished properly. Sub-marine gold does away with these annoyances. Dr. Herbst recommends to dip the pellet of gold to be used in water so as to make its introduction more easy, rendering the filling more dense. "I will make a personal remark," says Dr. Heide; "this way of filling resembles very much to the plugging of soft gold with pointed pluggers; but the finishing is done with burnishers applied on dental engine, then finished with corondum wheels. Dr. Herbst says that it is quite easy to add sub-marine gold to any fillings that are defective. The interesting point in this method, which is about the same as others in the way of filling with gold, is this: The peculiar quality of this gold, the limited number of instruments used for inserting a filling, is certainly a credit to Dr. Herbst, the eminent practitioner that every one has heard of—M. R. HEIDE, *L'Odontalgie.*

RONTGEN RAYS.—Referring to the great discovery of Professor Rontgen, and the applicability of the rays to gunshot injuries, and the impaction of foreign bodies, the editor remarks: "From the considerable difficulty experienced in adjusting the apparatus to the mouth the results, so far as the teeth are concerned, have perhaps not been quite as perfect as they were expected to be, still a good deal has been done. This suggests the value of the method of examination in those cases in which the unerupted bicuspid is removed to allow the incisors to fall back—an operation sometimes attended with considerable difficulty when the bicuspid is abnormally placed to the second temporary molar. The production of a photograph, such as shown by Mr. Harrison (at the meeting of the Midland Counties Board of the B. D. A.,) would be a valuable preliminary to the operation. Owing to the difference between the density of the tooth issues and the open bony tissue of the alveolar portion of the jaws, the teeth stand out in Rontgen photographs quite clearly from the surrounding bone, but usually—especially in the upper jaw—show a considerable amount of distortion. As far as methods are concerned, we believe the best results have been obtained by using a sensitive film in the mouth rather than a glass plate, the film, of course, incased in some light, tight material impervious to moisture, being capable of firm adaptation to the mucous membrane of the mouth. We cannot but think that there is still a future for this method of diagnosis in dental surgery, and with the more perfect knowledge of the nature of these mysterious rays, which time will assuredly bring, many of the difficulties attending the production of the radiograph will be removed."—*Journal of British Dental Association.*

---

THE CARE OF VULCANIZERS.—In spite of the fact that vulcanizers sold by reliable manufacturers are submitted to severe tests by hydrostatic pressure—some of them to a pressure of nine hundred pounds to the inch—explosions have not been unfrequent, generally due to the ignorance of the assistants who watch them, or pretend to watch them, during the process of vulcanization, and not a little to the carelessness of the dentists themselves, who fail to impress upon the students that these little steam boilers require as much attention as boilers of a greater capacity. In the first place, it does not pay to use cheap vulcanizers any more than cheap German tools and instruments, or the cheaper grades of artificial teeth. In the next place, one should familiarize himself with every part of the machine : realize the importance of keeping it and its belongings clean, and adhere strictly to the rules laid down by the manufacturers. Where thermometers are used it ought not to be forgotten that too rapid and too great heat at starting, especially if the flame is allowed to surround and reach the top cover, may deceive, and that the thermometer at 320° may really indicate only the temperature of the cover, and not that of the flask inside. The safety disk used on some modern vulcanizers should not be forced on too tight, as this weakens them. The boiler should on no account ever be perfectly full of water: at least, one inch or more of steam room should be left above the water, When the heat is first applied, the valve for the escape of steam should be opened for a few minutes to allow a free escape of steam, as this leaves in the boiler, after the valve is closed, an atmosphere of pure steam. and precaution should be taken not to use any more force in closing the valve than is necessary to make it steam-tight. We are warned, too, not to daub too much blacklead or soapstone powder about the packing. In fact, it is better never to use any if it can be avoided. Oil should never be used, as it rots the rubber packing, and may become gummy and cement the core to the pot, to the damage of the rubber packing. Too frequent use of the blacklead and soapstone wears away the screw thread. In heating up, the flame should not be larger than will cover the bottom of the boiler. If students can have these simple instructions put into their brains, it may save some of them from having their heads blown off. If they will not attend to them they deserve to be killed. Thermometers, in the cities, ought to be relegated to the list of things which are obsolete in the laboratory. Proper gas regulators and steam gauges should be used, and dentists owe it to themselves and their students not to neglect every proper precaution in the use of these laboratory demons.—*Dominion Dental Journal.*

VALUE OF MEAT EXTRACTS.—Professor Von Voit, of Munich, Germany, has investigated the extracts of meat and announces that such extracts have very little nutritive value, if any, and that their action is almost entirely a stimulating one, being due to their contents of alkaloids, such as creatin and creatinin.

---

BLOOD STAINS.—The statement has been lately published that a warm solution of tartaric acid is most efficient in removing blood stains from towels, aprons, etc. Acetic acid has long been in use for this purpose, and either acid will probably answer equally well.

---

HINTS.—By a Lazy Man.—1. You can buy prepared square cakes of pumice stone which are very convenient for cleansing the hands, polishing dirt off tables, and softening the stiff bristles on your chin before shaving. Only be sure and get it all off your chin before using soap and your razor.

2. After removing the bolts of your flasks, dip the screw in a bottle of oil convenient for the purpose, and they will always be easy for use again.

3. A first-rate laboratory bench-block may be improvised by securing on the table, one of the rubber-tipped door stops sold for the purpose of keeping doors from damaging the wall. They can be screwed into the table anywhere and in any position.

4. If your hands are dirty you will dirty rubber in packing sets, and the dirt remains, and even seems to penetrate deeper than the surface you wish to polish. The pumice block is a good thing to clean solid rubber before you pack.

5. A common soup ladle with holes made in the bottom to let the water out, is useful to lift hot flasks out of hot water.

6. A small hoe, used to scale fish, is useful for scraping hard plaster from the bench.

7. Articulators and head-rests are two articles which the capacious brains of our mechanics should improve.

8. I never need rubber scrapers—hardly ever. If you prepare your pattern plate properly, and are not too mean to buy well made pattern wax, your plate should come out ready for the sand-paper.

9. Do not use oil or shellac varnish to keep the second part of the plaster from sticking to the first in filling flasks. Keep a pot of the soap suds; after soaping the plaster, run water over it. It will not stick.

10. You can buy at any hardware store a neat, round and cheap asbestos plate on which to put rubber when you are packing. Clean and convenient.

11. You can buy in rubber stores, rubber fingers, which are useful if you have to finger foul mouths, and are just the thing to have on the finger of the hand which you use in the mouth when keeping the lips and tongue out of the way in extractions.

12. If you use beeswax or compound, have it ready rolled in thin sheets to save time.

13. Sulphate of potash always kept uncovered loses its value for hardening plaster.

14. If you insist upon placing a porcelain crown, either with or without a collar, upon a very hollow root with an opening larger at the neck than towards the apex, do not use amalgam as a retaining cement around the metal pivot. Thoroughly dry the root cavity; wipe it out finally with the merest suggestion of oil of cazeput, then pack it tightly with warmed gutta-percha—not the pink used for pattern plates—heat the metal pivot; work it into the gutta-percha, packing as well as you can. I prefer it to anything else.

15. After operating for patient afflicted with pulmonary tuberculosis, sterilize your instruments, hands, etc., as carefully as if you had been operating for a patient suffering from syphilis.

16. Some of the trouble which occurs in the vulcanizing of dental caoutchouc, is occasioned by a hap-hazard way of raising and retaining the temperature. If the directions advise you to keep the temperature at 315 degrees Fahr. for one hour and a quarter, it is a mistake to keep it at 320 degrees for an hour. In the matter of proper vnlcanizing, the slow and sure method in heating and cooling is the best.

17. If you buy a new drug, do not use it until you know all that is worth knowing about its action and uses, and if a a poison, its dangers and its antidotes.

18. A properly adjusted Auer light is away ahead of any electric light, if you are obliged to use artificial light at the operating chair. It is nearer the natural light of day than any other.

19. Keep your dental engine, chair and spittoon covered from dust at night. Do not use too much oil. Dirt is the bane of the dentist.

20. If you have rough hands from handling flanks, etc., wash well with hot water and soap when going to bed, rub vaseline on them thoroughly, and pull on a pair of large sized gloves.

Too lazy to say more just now.—*Dominion Dental Journal.*

---

EXPERIENCE.—By L. D. S.—He is a wise young man who is eager to profit by the experience of his seniors. Experience is a thing which cannot be found in books, or even obtained altogether from the experience of our seniors. One must get the most of it for himself. It is not an article that he can wholly transmit to others. It is a part and parcel of his own individual existence; as much as his own breathing and sleeping and eating. In trade and commerce, politics and the profession, it is a commodity of commercial value. which is reverenced and respected by wise and thoughtful men.

In the December issue editorial comment was made upon the want of sound judgment by many whose practical skill may be superior. It opened to my mind, as an old practitioner, a whole flood of recollections past and present, in my own career and that of others, and brought back vividly to me many a disappointing moment. Any one who ever witnessed the eminent and aged Dr. W. H. Atkinson, with his double pair of spectacles, performing the most exquisite operations, or who is familiar with the skilful work and sound judgment of the older school of operators in Europe and America, must appreciate the vigor of mind and body which makes such work possible. One does not find men of experience risking the wreck of reputation upon the wholesale insertion of crowns and bridges, and indulge in the fantastic fads —or frauds—of many of the junior practitioners. Perhaps I should not call them "frauds"; generally they do not know any better; and knowledge is the result of experience. A man of experienced practice can readily acquire any new practical idea which presents itself. But his experience helps him whether to accept or reject. And the judgment necessary in the latter is quite as valuable as in the former. We English people would be all the better for a little more of the Chinese reverence for age and experience.—*Dominion Dental Journal*

---

INGRATITUDE.—We delight in calling ourselves a scientific profession. But the bold facts of the case are, that while we have done very much in the applied sciences, our real scientific work has been confined to a very few individuals, who have mainly had their labor for their pains. The two most conspicuous examples of this are Miller and Black. The former ruined his health and sacrificed everything that usually makes life attractive, in his pursuit of scientific investigation for the benefit of his fellow practitioners. He published the cream of his discoveries in a book, the half of the first edition of which yet rests on the publisher's shelves. Men give him any amount of cheap applause, but they decline to spend two dollars in purchasing the record of his work.

Black has written a number of works of merit, and yet not one per cent. of the dentists of America have even a superficial acquaintance with what he has done. The only remuneration that he has ever received has been from the colleges which, in the estimation of those whose work consists in words instead of deeds, have done so much to debase dentistry. That is the way we of America too often foster and encourage scientific investigation. That is the way we are inclined to pay the debts incurred to those who work instead of talk. And it is a singular fact that those who talk the most have done the least. But after all, that is nothing strange. It is the usual way of the world.—*Dental Practitioner and Advertiser*.

